# Response to Phosphorus Limitation Varies among Lake Populations of the Freshwater Snail *Potamopyrgus antipodarum*

**DOI:** 10.1371/journal.pone.0085845

**Published:** 2014-01-16

**Authors:** Amy C. Krist, Adam D. Kay, Katelyn Larkin, Maurine Neiman

**Affiliations:** 1 Department of Zoology and Physiology and Program in Ecology, University of Wyoming, Laramie, Wyoming, United States of America; 2 Department of Biology, University of St. Thomas, St. Paul, Minnesota, United States of America; 3 Department of Biology, University of Iowa, Iowa City, Iowa, United States of America; Texas Tech University, United States of America

## Abstract

Local adaptation – typically recognized as higher values of fitness-related traits for native *vs*. non-native individuals when measured in the native environment - is common in natural populations because of pervasive spatial variation in the intensity and type of natural selection. Although local adaptation has been primarily studied in the context of biotic interactions, widespread variation in abiotic characteristics of environments suggests that local adaptation in response to abiotic factors should also be common. *Potamopyrgus antipodarum*, a freshwater New Zealand snail that is an important model system for invasion biology and the maintenance of sexual reproduction, exhibits local adaptation to parasites and rate of water flow. As an initial step to determining whether *P. antipodarum* are also locally adapted to phosphorus availability, we examined whether populations differ in their responses to phosphorus limitation. We found that field-collected juvenile *P. antipodarum* grew at a lower rate and reached an important size threshold more slowly when fed a relatively low *vs*. a relatively high- phosphorus diet. We also detected significant across-population variation in individual growth rate. A marginally significant population-by-dietary phosphorus interaction along with a two-fold difference across populations in the extent of suppression of growth by low phosphorus suggests that populations of *P. antipodarum* may differ in their response to phosphorus limitation. Local adaptation may explain this variation, with the implication that snails from lakes with relatively low phosphorus availability should be less severely affected by phosphorus limitation than snails from lakes with higher phosphorus availability.

## Introduction

Spatial variation in the strength and type of natural selection can generate local adaptation, whereby resident individuals possess higher relative fitness than individuals from other populations [1], [2]. Biotic interactions have been a particular focus of studies of the presence and strength of local adaptation, and these studies have shown that host-parasite interactions (e.g. [3]), plant-herbivore interactions (e.g. [4]), and predator-prey interactions (e.g., [5]) are all common sources of local adaptation. While abiotic factors as a driver of local adaptation have received less empirical attention, local adaptation has been documented with respect to factors such as geographic variation (e.g., [6]), latitudinal gradients (e.g., [7]), temperature (e.g., [8]), and soil type [9].

Here, we consider whether the New Zealand freshwater snail *Potamopyrgus antipodarum* possesses local adaptation to phosphorus availability. *Potamopyrgus antipodarum* is an important model system for the study of sexual reproduction (e.g., [10]) and invasion biology (e.g., [11], [12]). This snail exhibits local adaptation to trematode parasites among lakes [13], [14], [15], and among lake habitats [16], [17], and it also shows local adaptation in shell form among habitats differing in flow rate [18], [19], and predation by fish [18]. Invasive *P. antipodarum* also show population-specific responses in growth rate to different temperatures [11] suggesting local adaptation to temperature regime. The presence of distinct across-lake population genetic structure in *P. antipodarum* [20] increases the likelihood that across-population variation in the strength, direction, and/or efficacy of selection would generate local adaptation.

There is accumulating evidence that phosphorus availability is an important component of *P. antipodarum* ecology. Phosphorus often composes a relatively large fraction of organismal dry mass and is critical to growth and reproduction because the production of nucleic acids requires a relatively high input of phosphorus [21]. Two recent studies have demonstrated that growth in juvenile *P. antipodarum* is severely reduced by limited phosphorus [22], [23]. One of these studies also demonstrated substantial genetic variation in the extent to which growth is reduced by low availability of phosphorus [23], indicating that there is heritable variation for response to phosphorus limitation in *P. antipodarum*.

New Zealand lakes vary considerably in total phosphorus content [24] and phosphorus limitation of benthic algae (the main food source of *P. antipodarum*) differs among lakes (Krist et al., unpublished), leading us to hypothesize that *P. antipodarum* might be locally adapted to the availability of phosphorus in its native New Zealand. In particular, we predict that local adaptation would reflect selection favoring phenotypes that minimize consequences of phosphorus limitation in low-phosphorus environments [25].

To address whether *P. antipodarum* is adapted to local levels of phosphorus availability, we must first establish whether New Zealand populations differ in their responses to phosphorus limitation. To do this, we manipulated phosphorus content in the diets of juvenile *P. antipodarum* sampled from three New Zealand lakes. Because New Zealand *P. antipodarum* feature extensive ploidy variation [26], and because ploidy level can affect response to phosphorus limitation through the material costs associated with producing phosphorus-rich nucleic acids [23], [25], we restricted our study to triploid individuals.

## Materials and Methods

*Potamopyrgus antipodarum* were collected from rocks and aquatic vegetation with kick nets in January 2011 in the shallow regions of three New Zealand lakes (Brunner, Hawdon, Selfe) that have a high relative frequency of triploid snails. *Potamopyrgus antipodarum* is not an endangered or protected species, and necessary permits were granted by the New Zealand Department of Conservation and the Iowa Department of Natural Resources. The triploid component of lake populations of New Zealand *P. antipodarum* generally harbor very high genetic diversity [27], [28], [29], meaning that even a single lake collection likely included many distinct triploid genotypes. After collection, all snails were transported to the University of Wyoming, where the experiment was conducted.

All of the snails in the experiment were <2.5 mm in shell length at the beginning of the experiment, below the 2.5–3.0 mm shell length threshold typically used to designate adult *vs*. juvenile *P. antipodarum* (e.g., [30], [31]). We used as many juvenile snails as were available from each lake, which ranged from 85 individuals (Brunner & Hawdon) to 96 individuals (Selfe), for a total of 266 snails at the start of the experiment.

From each lake, individual snails were randomly assigned to either the high or low phosphorus (“high P” or “low P”) diet treatment, such that about half of the snails from each lake sample were fed each diet treatment. We produced low-P (carbon:phosphorus ∼984; standard deviation (SD) for % carbon = 2.41, % phosphorus = 0.04) and high-P (carbon:phosphorus ∼239; SD for % carbon = 1.38, % phosphorus = 0.09) diets for *P. antipodarum* by manipulating the carbon:phosphorus ratio of the green alga *Scenedesmus obliquus*. The molar carbon:phosphorus ratios of the experimental diets were above (low P) or below (high P) the threshold elemental ratio [32] for phosphorus limitation, estimated at carbon:phosphorus ∼270 for *P. antipodarum* [22]. We have already demonstrated that growth of juvenile *P. antipodarum* is severely reduced by low-P *S. obliquus*, indicating that this diet treatment does cause phosphorus limitation in *P. antipodarum* [22], [23].

We fed each snail 0.0035 g dried algae (re-suspended in 1 ml of well water), equivalent to *ad libitum* food levels for juvenile *P. antipodarum* (high-food level in [33]), 3 times per week. We measured shell length of all individuals every two weeks using an ocular micrometer on a dissecting microscope (Leica S6E). Snails were housed individually in round plastic cups (300 ml). We changed the water in each cup 2 times per week and cleaned the cups once a week. We housed the snails at 18°C on a 12-hour light and 12-hour dark cycle. After 81 days of the diet treatments, a similar duration to previous studies that have demonstrated clear effects of diet treatments on growth and reproduction in *P. antipodarum* (e.g., [34], [35]), we transferred all snails to the University of Iowa. We then measured final shell length to the nearest tenth of a millimeter under a dissecting microscope.

The New Zealand lakes we sampled contain both diploid sexuals, which are ∼50% males [36], [26], and polyploid asexuals, which are ∼2–5% males [26], [37]. Because ploidy level can affect response to dietary phosphorus [23] and because male and female *P. antipodarum* grow at different rates (Neiman, unpublished data), we restricted our analysis to triploid females. However, because juvenile snails (<3.0 mm in shell length) cannot be sexed reliably (e.g., [38]) and because ploidy determination in *P. antipodarum* requires sacrificing the individual (e.g., [26], [37]), we had to wait until the end of the experiment to sex each snail and determine ploidy. At this time, we determined the sex of each snail using presence (male) or absence (female) of a penis for each snail >3.0 mm in shell length. We then dissected each snail and removed and snap-froze head tissue of all snails without penises (females and juveniles) for ploidy determination via flow cytometry.

Following Neiman et al. [26] [37], we prepared each sample for flow cytometry by grinding frozen head tissue in a solution containing 0.2 M Tris-HCl (pH 7.5), 4 mM MgCl_2_, 1% TritonX-100, and 4 ug/mL DAPI. This solution was filtered through a 70-micron nylon sheet and then run on a Becton Dickinson LSR II flow cytometer. We used the FL1 channel to assess the DAPI fluorescence (and thus the DNA content) of sample nuclei under a UV lamp. At the beginning and end of each flow cytometry run, we calibrated the machine with 20 µL of chicken red blood cells (Lampire Biological Labs, Pipersville, PA) treated and filtered as for the snail head tissue. We adjusted the gain of the flow cytometer so that the chicken standard peak was always centered on 80 FL1 units. Each standard and sample was run until a count of 10,000 events was achieved. We analyzed all results using FlowJo software (Version 8.8.7, Tree Star, Inc.).

We first used FlowJo software to confine data analysis for each flow cytometry sample to the peak of data points corresponding to intact nuclei of single cells in growth phase 1. We calculated the mean fluorescence for this peak region for each sample and standardized this mean by dividing it by the mean FL1 value of the chicken red blood cell standard used to calibrate that particular run. Because there is substantial population-level variation in genome size even within ploidy level [26], we used a histogram of the distribution of these standardized FL1 values for each sample within each population to assign ploidy status. As previous analyses of ploidy in *P. antipodarum* have demonstrated [26], [37], [39], flow cytometry readily distinguishes among diploid, triploid, and “tetraploid” (>3×) *P. antipodarum*.

We were able to use flow cytometry to determine the ploidy of all of the 266 snails in the experiment. Because we were focused on triploids, we excluded the 6 tetraploids and 83 diploids from subsequent analyses. We also omitted the one triploid male that we identified (out of the 88 adult triploid individuals sexed) for a total of 176 triploid individuals in our final dataset. Of these 176 triploid snails, 87 were >3.0 mm in shell length and had been confirmed by the absence of a penis as adult females; 57 snails were <3.0 mm in shell length, and were thus classified as juveniles. The other 32 snails were >3.0 mm in shell length but due to an unintended oversight were left unsexed. However, the overwhelming predominance of females (87 of 88 snails; 98.9%) amongst the adult triploids that we sexed means that all or nearly all of both the unsexed adult and juvenile triploid snails were also females (also see [26], [37]). We thus included all 176 of these snails in subsequent data analyses.

Paired samples t-tests indicated that there were significant (*p*<0.05) increases in shell length between each consecutive set of shell length measurements (*p*<0.002 for all comparisons; Fig. S1). Because this meant that snails were still experiencing rapid growth at the conclusion of the experiment, we used the total increase in shell length between the 81 days marking the beginning and end of the experiment as our primary response variable.

We used ln(final shell/initial shell length))/81 days to estimate specific growth rate (SGR) for each snail. We then used a two-way univariate ANOVA to determine whether the fixed factors of diet treatment and lake alone or in interaction affected specific growth rate and conducted Tukey posthoc pairwise comparisons to determine whether there were differences in SGR among lakes. We used a Fisher's exact test to compare the proportion of snails in the low-P *vs*. high-P treatments that crossed the 3.0 mm shell length threshold typically used to assign “adult” status in *P. antipodarum* (e.g., [30], [31]). All statistical tests were implemented with IBM SPSS Statistics (version 21.0) with the exception of the Fisher's exact test, for which we used http://graphpad.com/quickcalcs/contingency1.cfm.

## Results

**Figure 1:**
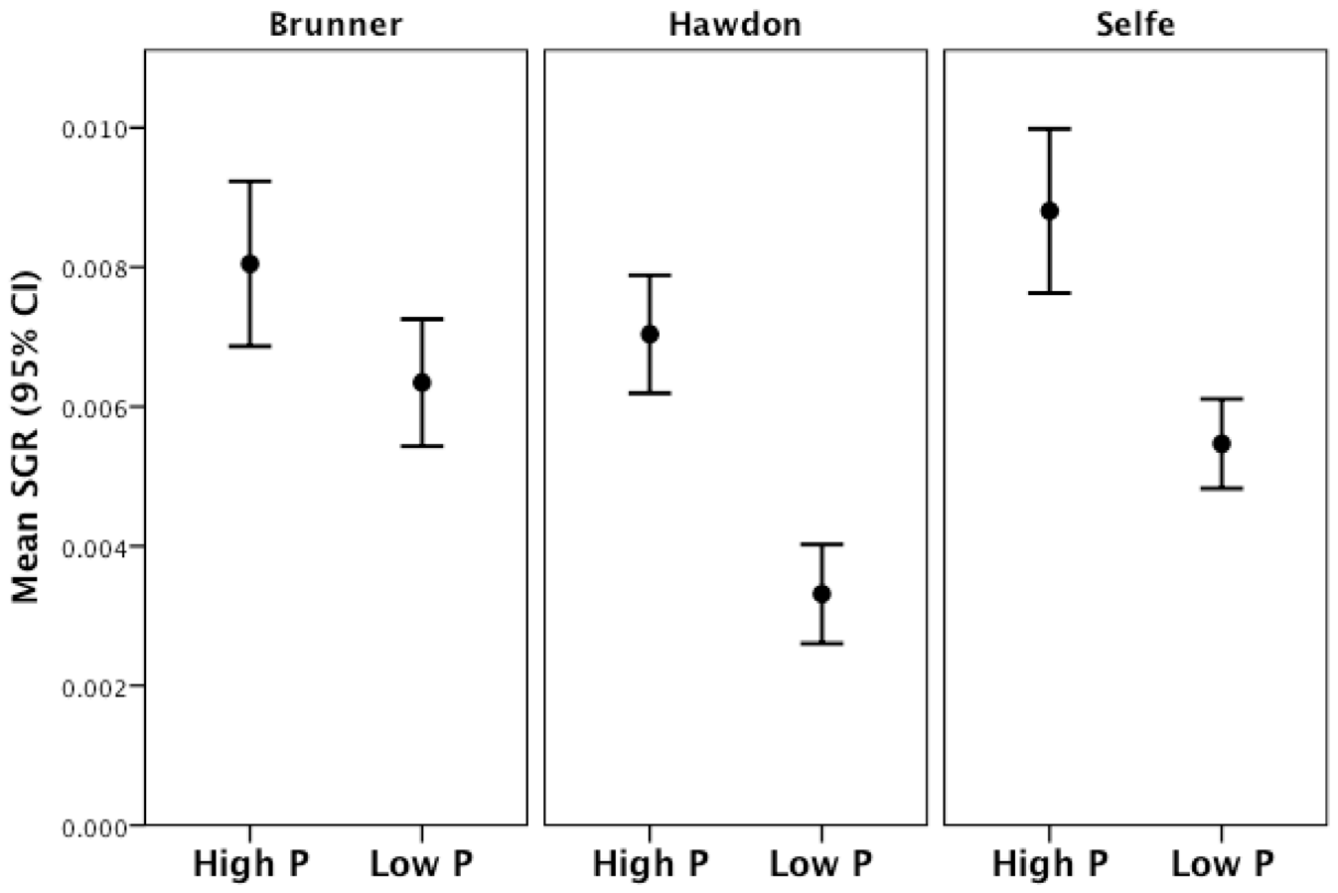
Mean SGR of snails in the high-P *vs*. low-P treatments. Error bars are 95% confidence intervals. *Potamopyrgus antipodarum* fed the low-P diet grew at a significantly lower rate than snails fed the high-P diet. There were also significant differences in SGR among lakes, driven by significantly higher SGR in Brunner (N = 41) and Selfe (N = 54) snails relative to Hawdon (N = 81; *p*<0.001 for both comparisons) and a marginally significant lake by diet treatment interaction (*F*_2, 170_ = 2.52, *p* = 0.083).

**Figure 2:**
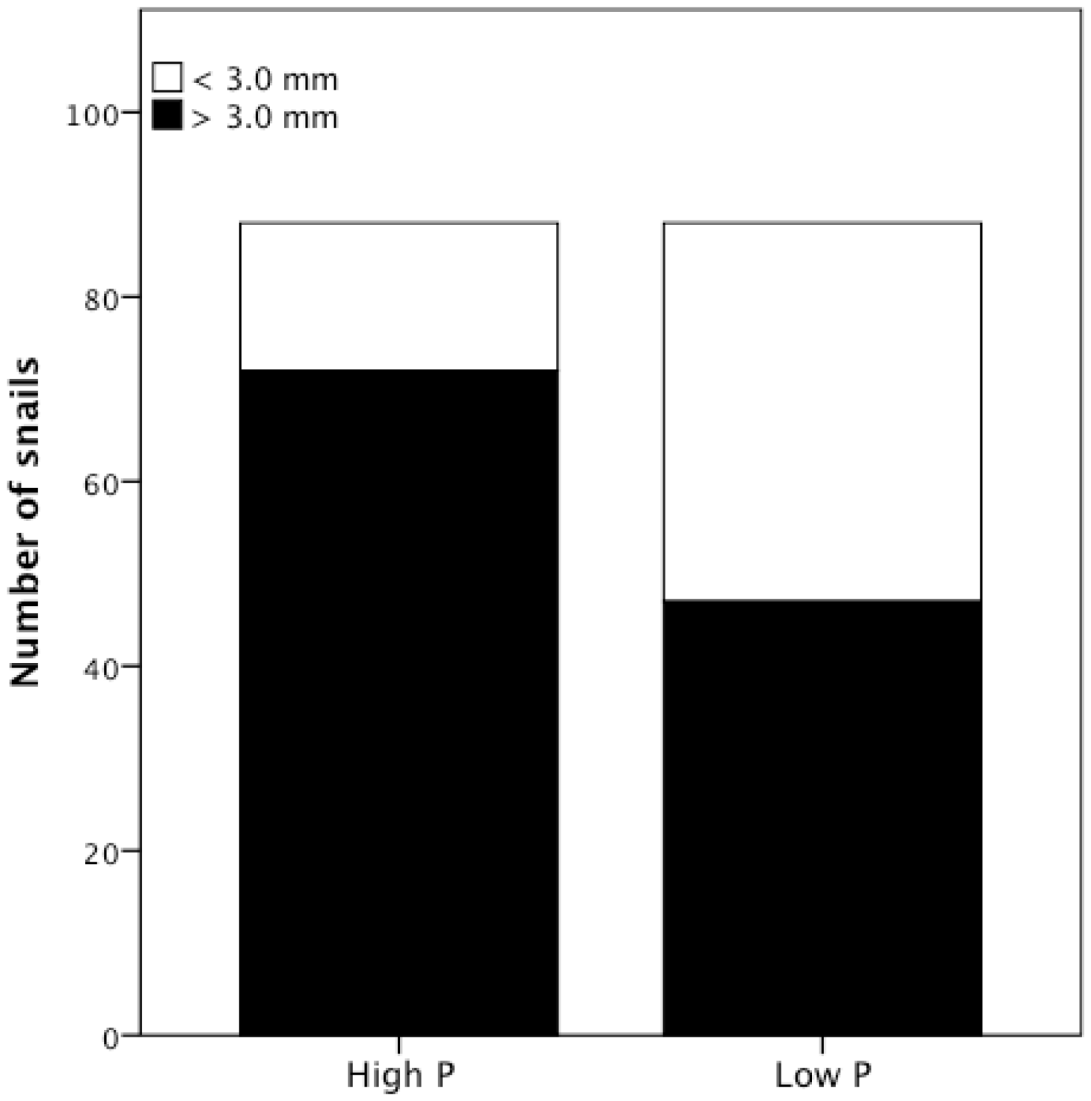
More snails attained the 3.0*P. antipodarum* in the high-P *vs*. low-P diet treatment (Fisher's exact test, *p*<0.0001).

Diet treatment had a large effect on SGR of *P. antipodarum* (*F*_1, 170_ = 71.23, *p*<0.0001; Fig. 1), such that growth was ∼64% higher in snails fed the high-P diet (mean SGR = 0.008+/− 0.003 mm SD) than the low-P diet (mean SGR = 0.005+/− 0.002 mm SD). Significantly more snails attained the 3.0 mm shell length threshold in the high-P diet *vs*. the low-P diet treatment (Fisher's exact test, *p*<0.0001; Fig. 2), suggesting that the lower rate of growth experienced by *P. antipodarum* in the low-P diet treatment might also delay reproductive maturity (e.g., [22]). Taken together, these data indicate that snails receiving the low-P diet experienced phosphorus limitation. Lake of origin also significantly affected SGR of snails (*F*_2, 170_ = 14.73, *p*<0.0001; Fig. 1). Posthoc pairwise Tukey comparisons indicated that this effect of lake was largely driven by the high SGR of snails from Lakes Brunner and Selfe relative to snails from Lake Hawdon (Fig. 1).

We also found a marginally significant lake of origin by diet treatment interaction (*F*_2, 170_ = 2.52, *p* = 0.083). Although we lacked statistical power to detect a significant interaction because of limited sample sizes in Lake Brunner [40] (20 and 21 snails per treatment in Lake Brunner, power = 0.60), Fig. 1 reveals large differences in the effect of the high-P *vs*. low-P diet on SGR among snails from different lakes. We thus compared the magnitude of the decrease in SGR from the high-P relative to the low-P diet for *P. antipodarum* across lakes, which represents the response of each population to phosphorus limitation. We found that the largest differences in SGR between the high-P and low-P diets were in snails from Lakes Hawdon and Selfe (Hawdon; mean SGR on high-P diet = 0.007, low-P diet = 0.003: Selfe; high-P diet = 0.009, low-P diet = 0.005) and the smallest difference was in snails from Lake Brunner (high-P diet = 0.008; low-P diet = 0.006; Fig. 1). This means that snails from Lakes Hawdon and Selfe experienced an approximately twofold increase in the severity of the response to phosphorus limitation relative to snails from Lake Brunner.

## Discussion

Juvenile *P. antipodarum* fed a low-P diet grew at a substantially lower rate and reached an important body size threshold more slowly than juveniles fed the high-P diet. These growth rate responses likely translate into fitness differences because *P. antipodarum* females that grow more slowly reach reproductive maturity later than females that grow more rapidly [22] and because fecundity and the rate of achieving reproductive maturity are both positively correlated with body size in female *P. antipodarum* [22], [41], [42].

This result is the first evidence for phosphorus limitation in field-collected *P. antipodarum*. We also demonstrated population-level variation in specific growth rate, and detected preliminary evidence that the sensitivity of *P. antipodarum* to phosphorus limitation may vary across lakes. This potential difference in response to phosphorus limitation may result from snails being locally adapted to the availability of phosphorus in their lakes of origin.

While studies measuring across-lake phosphorus availability and reciprocal transplant experiments between lakes that differ in phosphorus availability are needed to conclusively establish the presence (or absence) of local adaptation to phosphorus availability, the particularly severe responses of *P. antipodarum* from Lakes Hawdon and Selfe to limited phosphorus relative to Lake Brunner suggests the existence of the type of across-lake variation in sensitivity to limited phosphorus that would be expected under local adaptation. One testable prediction that follows from our results is that, under local adaptation, the availability of phosphorus in the diet of *P. antipodarum* from Lakes Hawdon and Selfe should be higher than for snails from Lake Brunner.

Evidence for other types of local adaptation in *P. antipodarum* (e.g., parasites [13]–[17], flow rate [18], [19], temperature [11]) combined with marked across-lake population structure of *P*. *antipodarum* in its native New Zealand [20] and substantial genetic variation in the extent to which growth of *P. antipodarum* is reduced by low availability of phosphorus [23] suggest that local adaptation to phosphorus availability is a plausible explanation for the variation in response to phosphorus limitation among lake populations of *P. antipodarum* that we found. In fact, our finding that SGR varies among lakes, despite the generally high diversity of asexual lineages that are expected within each lake, suggests that SGR may differ among lakes because of local adaptation.

Evidence for across-population variation in the response of *P. antipodarum* to phosphorus limitation is relevant to evaluating a hypothesis suggesting a potential connection between sensitivity to phosphorus limitation and the outcome of competition between *P. antipodarum* differing in ploidy level and/or reproductive mode [23], [25], [43]. In particular, we hypothesized that *P. antipodarum* with higher ploidy level should be more sensitive to phosphorus limitation because of the material cost of producing extra nucleic acids, which are rich in phosphorus [25], [43]. In support of this hypothesis, we found that relative to triploids, tetraploid *P. antipodarum* suffered a much larger reduction in growth on a low-P relative to a high-P diet [23]. Because sexual *P. antipodarum* are diploid and asexuals are polyploid, this hypothesis may also contribute to understanding the distribution and maintenance of sexual reproduction. The next steps in evaluating this hypothesis require establishment of spatial variation in dietary phosphorus availability and in response of *P. antipodarum* to limited phosphorus. The data presented here provide preliminary evidence of the latter and set the stage for future studies that provide more rigorous tests of local adaptation to phosphorus availability in New Zealand populations of *P. antipodarum*.

## Supporting Information

Figure S1 **Mean SGR of snails in the high-P (closed circles) and low-P diets (open circles) for each population over 6 two-week intervals during the 81 days of the experiment.** Error bars are 95% confidence intervals. SGR for snails from Lake Hawdon are missing for 4/26/2011 because snails were accidentally not measured on this date.
(EPS)
